# Supplementary material for: Predicting visual function by interpreting a neuronal wiring diagram
Source: Nature. 2024 Oct 2;634(8032):113–23. doi: 10.1038/s41586-024-07953-5 (PMC11446822; doi:10.1038/s41586-024-07953-5)
Supplement: Supplementary file 6 — CRF and ERF predictions for individual TmY4 and TmY9 cells. Analogous to Supplementary Data 3, but for TmY target types. Shown are the top four monosynaptic pathways, the strongest pathway passing through each of the top ten intermediary types (ranking from Extended Data Fig. 7), and the trisynaptic pathway Tm1–TmY–Dm3–TmY (see the section entitled Prediction of spatial normalization). [file 41586_2024_7953_MOESM6_ESM.zip › DataS4/TmY9q/720575940613115545.pdf]

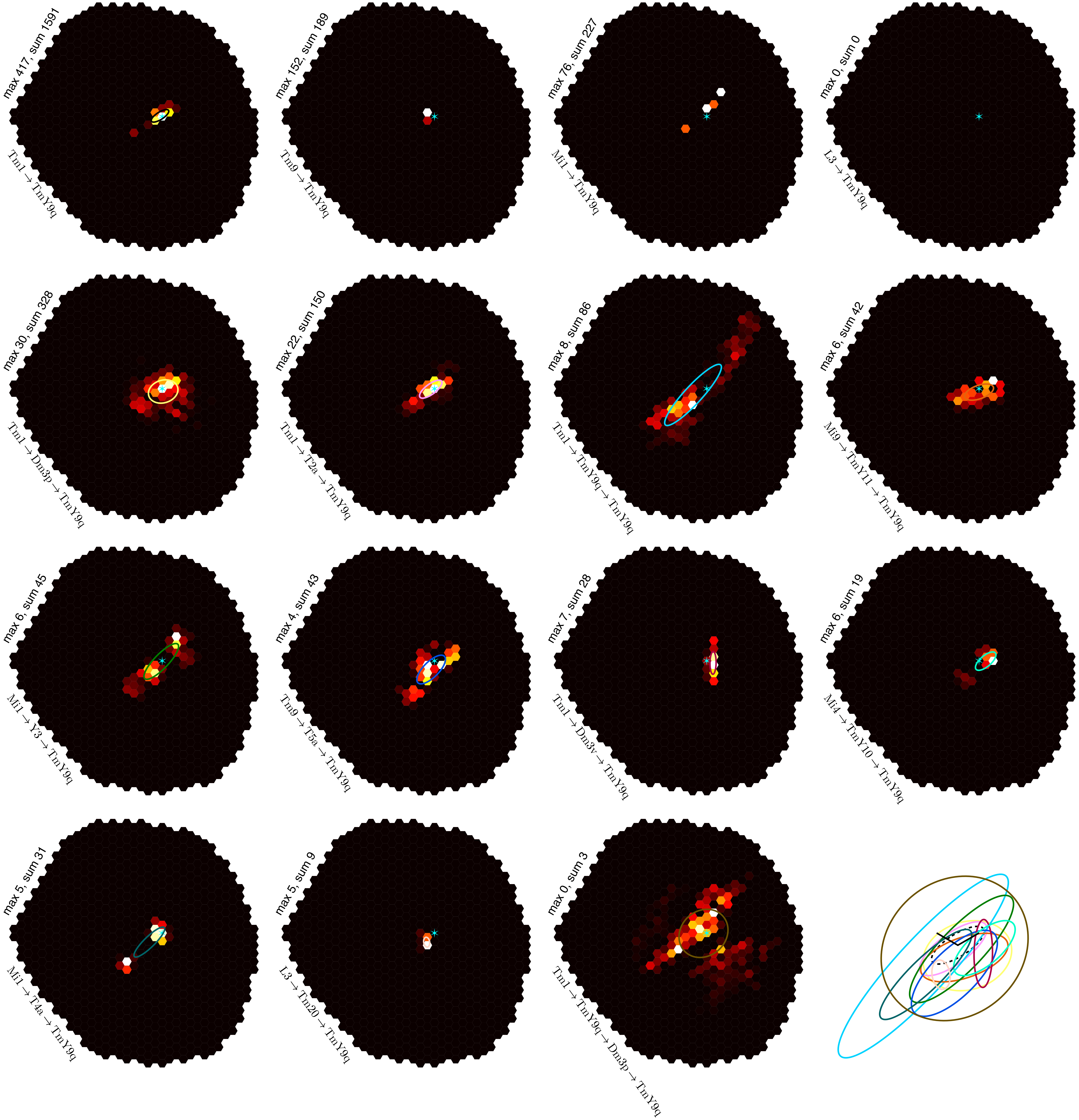

max 417, sum 1591
Tm1 → TmY9q
max 152, sum 189
Tm9 → TmY9q
max 76, sum 227
Mi1 → TmY9q
max 0, sum 0
L3 → TmY9q
max 30, sum 328
Tm1 → Dm3p → TmY9q
max 22, sum 150
Tm1 → T2a → TmY9q
max 8, sum 86
Tm1 → TmY9q → TmY9q
max 6, sum 42
Mi9 → TmY11 → TmY9q
max 6, sum 45
Mi1 → Y3 → TmY9q
max 4, sum 43
Tm9 → T5a → TmY9q
max 7, sum 28
Tm1 → Dm3v → TmY9q
max 6, sum 19
Mi4 → TmY10 → TmY9q
max 5, sum 31
Mi1 → T4a → TmY9q
max 5, sum 9
L3 → Tm20 → TmY9q
max 0, sum 3
Tm1 → TmY9q → Dm3p → TmY9q
